# The Archaeal Exosome: Identification and Quantification of Site-Specific Motions That Correlate with Cap and RNA Binding**

**DOI:** 10.1002/anie.201302811

**Published:** 2013-06-26

**Authors:** Maxime J C Audin, Georg Dorn, Simon A Fromm, Kerstin Reiss, Stefan Schütz, Matthias K Vorländer, Remco Sprangers

**Affiliations:** * Max Planck Institute for Developmental Biology, Spemannstrasse 35, 72076 Tübingen (Germany) Interfaculty Institute of Biochemistry, University of Tübingen(Germany)

**Keywords:** exosome, molecular machines, NMR spectroscopy, protein dynamics

Large molecular machines perform many cellular processes and it is of fundamental interest to understand how these enzyme complexes work in detail. In this regard, not only an accurate description of the static three-dimensional (3D) structure is required, but also a description of how these machines change their structure over time.

These internal protein motions have been shown to be important for, for example, biomolecular recognition,^1^ allostery,^2^ protein stability,^3^ and enzymatic activity.^4^ NMR spectroscopy is especially suited to study internal motions and when combined with methyl TROSY techniques^5^ is able to address this aspect in very large molecular assemblies.^6^

The exosome complex is a large molecular machine that degrades or trims different RNA substrates in the 3′ to 5′ direction.^7^ The archaeal and eukaryotic exosome complexes consist of nine subunits arranged in a hexameric ring structure (the exosome core) that interacts with a trimeric cap structure.^8^ In the archaeal exosome complex the core contains three Rrp41/Rrp42 heterodimers^9^ and the cap contains three copies of Rrp4 or Csl4 or a mixture thereof^10^ (Figure S1 in the Supporting Information). A number of crystal structures of the archaeal exosome have been solved that show that the RNA is funneled through a hole at the top of the exosome core.10a, 11 In isolation the hexameric 173 kDa exosome core is catalytically active, where binding of the trimeric Rrp4 (or Csl4) cap modulates both catalytic activity and substrate selectivity.^12^

Herein, we present methyl TROSY NMR experiments that address the potential dynamics of the exosome complex in solution. A prerequisite for detailed NMR studies of biomolecular structures, interactions, and dynamics is that high-quality spectra can be recorded. For large molecular machines this is challenging in several ways. First, significant signal overlap arises due to the high number of unique resonances. To simplify such spectra, we prepared exosome complexes that contain NMR-active Rrp42 in an otherwise NMR-inactive background. Second, fast relaxation rates in large complexes lead to substantial broadening of the NMR signals. To overcome this, we made use of methyl TROSY spectroscopy^5^ on samples that contained NMR-active Ile-δ1, Leu-δ, and Val-γ [^1^H,^13^C] methyl groups in an otherwise fully deuterated background (Figure 1 A and Figure S2).

**Figure 1:**
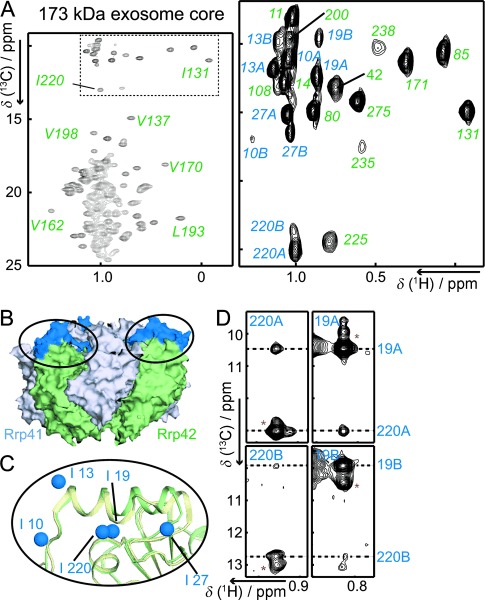
A) Methyl TROSY spectrum of the 173 kDa exosome core that contains NMR-active Rrp42. The boxed Ile-δ1 region is enlarged and residues that show two sets of peaks are labeled “*A*” and “*B*”. B) The region that adopts two conformations is colored blue on the surface of the exosome core. This region is part of the cap-binding interface (see also Figure S1). C) Enlargement of the indicated region in (B), where Rrp42 as found in the exosome core (green; PDB: 2BR2) is superimposed on Rrp42 and as found in the exosome-Rrp4 complex (olive; PDB: 2JE6). The helix in the Rrp42 cap-binding region adopts a different conformation upon interaction with the Rrp4 cap. The blue spheres show the positions of the exchanging Ile residues. Note the short distance between Ile 19 and Ile 220. D) Planes from a 3D (H)-C-C-H NOESY spectrum that displays interproton NOE contacts between Ile 19 and Ile 220 in both states, indicating that the structures of state A and state B in the free exosome complex are similar. Cross peaks indicated with a red asterisk result from chemical exchange between the two states (see Figure 2).

To assign the Ile-δ1, Leu-δ,and Val-γ methyl resonances of Rrp42 in the exosome core and exosome–cap complexes, we followed the divide-and-conquer approach (Figure S2A).6a To validate and complete the Rrp42 assignments in the exosome complexes, we took a mutational approach6c, 13 (Figure S2B, Table S1). In summary, we have assigned more than 70 % of the methyl groups in the exosome core complex. It should, however, be mentioned that 100 % of the fully resolved resonances that can be used to study interactions and dynamics were assigned.

The assignment of the methyl groups in the exosome core complex revealed that a subset of the residues gives rise to two sets of resonances (Figure 1 A), which indicates that the complex adopts two structurally different conformations in solution. In the following we will refer to the set of peaks that are more intense as state A and the other set of peaks as state B. It should be noted that the isolated Rrp42 (Figure S2A) adopts only a single conformation, implying that Rrp41 is required to induce the different states in the exosome complex. In the exosome core, the residues that display two states are clustered at the top of the core complex (Figure 1 B), a region that contains the entrance pore for the substrate and that is responsible for the interaction with the Rrp4/Csl4 cap complex (Figure S1).

There is no evidence for conformational variability in the free exosome core based on the high-resolution crystal structure of the complex.^9^ There, the unit cell contains four exosome complexes and all independent copies of Rrp42 superimpose with a backbone root-mean-square deviation (RMSD) of less than 0.13 Å. The only evidence for structural plasticity in the exosome core can be derived from the static structure of the exosome–cap complex. There, Rrp4 induces a small structural change in Rrp4210a in the region that we observe to be structurally inhomogeneous in the free exosome core (Figure 1 B,C).

To further characterize the two observed Rrp42 states we recorded methyl–methyl NOESY spectra. Interestingly, we observe the same methyl–methyl contacts for state A and state B (Figure 1 D), indicating that the two states have similar structures. To determine the rates and populations that are connected with the exchange process between states A and B, we used longitudinal exchange NMR experiments.6c, 14 In the exosome core, we could reliably extract exchange rates and populations for four isoleucine residues (13, 19, 27, and 220). As all four residues have very similar exchange rates we used one global fit to extract a single exchange rate and one set of populations (Figure 2 A). From this, we determined that the exosome core exchanges between state A and state B with a rate of 36.1±1.9 Hz and that the population of state A is 0.76±0.01 at 60 °C. To independently validate the extracted exchange parameters we used multiple quantum (MQ) relaxation dispersion experiments,^15^ from which we extracted an exchange rate of 44.24±26.8 Hz and a population of state A (pA) of 0.82±0.08 at 60 °C (Figure 2 B). These parameters are in agreement with the values obtained from the longitudinal exchange experiments and thus independently validate these data.

**Figure 2:**
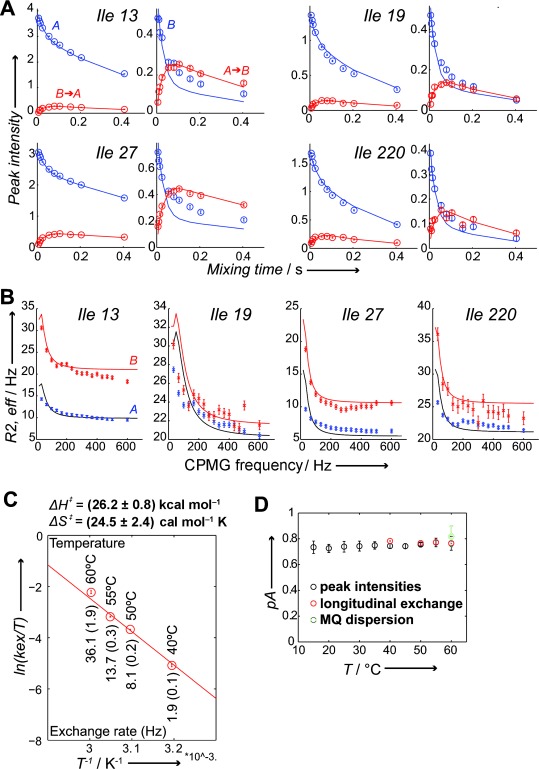
Quantification of the exchange process in the exosome core. A) Longitudinal exchange experiments. Auto and cross peaks are colored blue and red, respectively. Experimental data points are indicated with open circles, and the solid lines correspond to the best fits of the data. (All data are fitted simultaneously to one global exchange rate and one set of populations.) B) MQ dispersion data. The blue and red curves correspond to the states A and B, respectively. (All data are fitted simultaneously to one global exchange rate and one set of populations.) C) Eyring plot derived from kinetic data determined at four different temperatures. D) The population of state A does not change significantly over a large temperature range.

To access thermodynamic properties associated with the exchange process we measured exchange rates and populations at temperatures between 40 and 60 °C. Based on that, we derived an activation enthalpy of 26.2±0.8 kcal mol^−1^ and an activation entropy of 24.5±2.4 cal mol^−1^ K^−1^ for the conformational exchange (Figure 2 C). The positive value for the activation entropy indicates that the disorder in the transition state is greater than in the two ground states. If one assumes that the hydration does not change significantly during the exchange process, this implies that the Rrp42 N-terminal helix partially unfolds upon changing between states A and B. The magnitude of the activation entropy is similar to that observed for the aromatic ring flips in the core proteins^16^ and implies that the transition state is structurally distinct from the two ground states. Between 15 and 60 °C the populations of states A and B are almost invariable at pA=0.75 (Figure 2 D). This indicates that the two states have similar enthalpy, which is consistent with state A and state B being structurally similar (Figure 1 D). The small entropy difference also implies that state A (which is populated to a larger extent) is favored over state B due to increased entropy. In agreement with that, we observe lower order parameters (high flexibility) for two of four methyl groups in state A (Figure 3). Taken together, we have shown that the cap-binding region in the isolated exosome core complex exchanges between two states in solution. From our NOE and thermodynamic data we can conclude that the two conformations are structurally similar.

**Figure 3:**
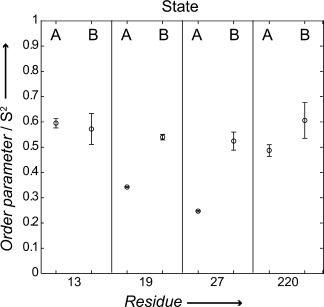
Order parameters for states A and B of Ile 13, 19, 27, and 220. State A is significantly more flexible for residues 19 and 27, suggesting that state A is more flexible than state B; this is in agreement with a higher expected entropy of that state.

To determine whether the motions in the free exosome are directly correlated with the cap-binding process, we prepared exosome samples in complex with NMR-inactive Rrp4 and Csl4. Interestingly, we noticed that the residues that show two conformations in the free exosome core complex only display a single set of resonances in the exosome–cap complex (Figure 4 A,B). This shows that the dynamics in the exosome core are significantly affected or even diminished upon interaction with the cap. To validate this observation, we recorded MQ dispersion experiments on the exosome–Rrp4 and exosome–Csl4 complexes that show that the residues that are dynamic in the free exosome core complex do not show micro- to millisecond time-scale dynamics in the cap-bound form (Figure S3). These observations are compatible with a scenario where one of the two states in the free exosome recruits Csl4 or Rrp4. It is, however, not straightforward to determine whether state A or state B interacts with the cap proteins. First, due to the chemical shift changes that Rrp42 experiences upon cap binding (Figure 4 A,B), we cannot directly deduce which state the exosome adopts when in complex with the cap. Second, the interaction between the cap and the exosome core is slow on the NMR chemical shift timescale, which prevents the determination of which of the two states is selected for by the cap in a titration experiment. Finally, chemical shifts that are predicted^17^ based on the two structures (free and cap-bound) do not correlate with the observed chemical shifts (A or B) in any of the possible combinations (Figure S4), likely reflecting difficulties in the accurate prediction of methyl groups' chemical shifts for large protein complexes.

**Figure 4:**
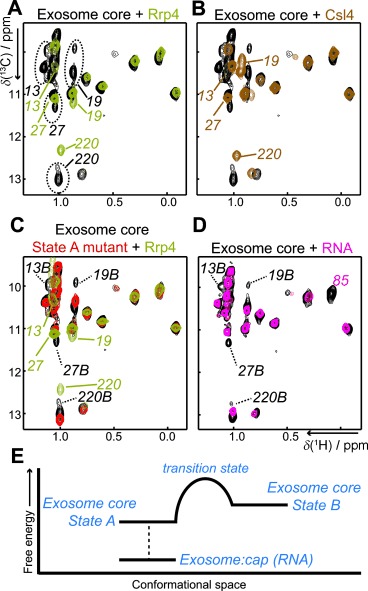
State A in the exosome core is stabilized upon interaction with cap proteins or with substrate RNA. (A, B) Spectra of the exosome core in the absence (black, dashed oval, two states) and presence of Rrp4 (A, olive) or Csl4 (B, brown). Upon cap interaction only a single state is observed in the exosome. The cap complexes interact with state A (see text), although this cannot be derived from the spectra directly due to the chemical shift changes induced by the cap. The signal to noise ratio for isoleucines 13, 19, 27, and 220 is larger than 10; thus, a potential minor state with a population of 25 % would have been observed in the spectra. C) Superposition of the WT exosome (black), the Rrp42 state A mutant where state B is no longer visible in the absence (red) and presence of Rrp4 (olive). In complex with Rrp4, Rrp42 is structured identically in the state A mutant exosome (olive) and in the WT exosome (Figure 1 A, olive spectrum) (Figure 4 A). D) Exosome core in the absence (black) and presence (pink) of an equimolar amount of RNA substrate (one RNA molecule per hexameric exosome complex). State B in the exosome core is no longer visible upon formation of the substrate–enzyme complex. In addition, residues close to the active site experience chemical shift perturbations (e.g Ile 85). E) Schematic representation of the reaction coordinates of the exosome core. States A and B have similar enthalpy; state A has a lower free energy due to increased entropy. The transition state is more disordered than the ground states A and B. State A interacts with the cap structure through a mechanism of conformational selection.

To experimentally address which state in the free exosome complex interacts with the cap, we designed a mutant exosome core in which the equilibrium between the two states is significantly altered. Interestingly, we found a point mutant that is remote from the cap-binding interface (Figure S5) that displays only state A in ^1^H,^13^C-HMQC spectra (N9A in Rrp42, referred to as “state A mutant”) (Figure 4 C, red). We then used this state A mutant to probe binding for the cap proteins; if this mutant efficiently interacts with Cls4 and Rrp4, this is strong evidence that state A is selected by the cap proteins. Interestingly, this is exactly what we observe. First, an NMR spectrum of the state A mutant in complex with Rrp4 (Figure 4 C, olive) is identical with the spectrum of the wild-type (WT) exosome in complex with Rrp4 (Figure 4 A, olive). Secondly, surface plasmon resonance (SPR) experiments confirm that the state A mutant still interacts strongly with Rrp4 (Figure S6). Thirdly, a reduced form of Rrp4, where one of the three protein domains is deleted, interacts stronger with the exosome of the state A mutant than with the WT exosome in NMR titration experiments (Figure S7). In summary, all binding experiments show that the exosome complex of the state A mutant is fully capable of interacting with the cap proteins. We thus propose that state A in the exosome complex corresponds to the cap-bound conformation. Our data are thus compatible with a model where the exosome–cap interaction occurs through a process where only one of the possible conformations is selected upon complex formation.

Upon addition of substrate RNA to the exosome core complex (Figure 4 D)^9^, 11a we observed significant chemical shift perturbations for Rrp42 residues Ile 85 and Val 86. Based on the structure of the *Pyrococcus abyssi* exosome in complex with RNA,^18^ these residues are indeed close to the substrate. In addition, we observe a significant shift towards state A for the residues at the cap-binding region. This indicates that substrate RNA, like the cap structure, interacts with state A in the free exosome complex (Figure 4 E).

In summary, we have complemented the known static crystal structure of the archaeal exosome core with quantitative information regarding unanticipated internal dynamics. Our data show that molecular motions often remain undetected in protein structures and underscore the importance of studies that address the localization, quantification, and interpretation of these functionally important aspects of large molecular machines. We anticipate that future work of the sort presented here will be able to address the relation between dynamics and function in many biomolecular assemblies.

## Experimental Section

NMR spectra were recorded between 15 and 60 °C on Bruker AVIII-600 and AVIII-800 spectrometers. All spectra displayed in the figures were recorded at 50 °C. Longitudinal exchange experiments were recorded as a series of 3D (C-C-H) data sets with mixing times between 0 and 800 ms. Exchange parameters were extracted as described.6c Errors in the measured data were determined based on the noise level in the spectra. The error in the extracted parameters is based on Monte Carlo simulations, where back-calculated data were randomly changed according to the experimental error. Methyl TROSY relaxation dispersion experiments^15^ were recorded as a series of 2D data sets using constant-time relaxation periods of 50 ms and CPMG (Carr–Purcell–Meiboom–Gill) frequencies ranging from 33 to 600 Hz. The dispersion data were fitted numerically as described, where the chemical shift differences were extracted directly from the spectra.6c Errors in the parameters were based on Monte Carlo simulations and on duplicate measurements. Uncertainties in the extracted chemical shift differences were accounted for by varying **Δω**H and **Δω**C by 0.005 and 0.01 ppm, respectively. Methyl group order parameters were determined as described,^19^ using a rotational correlation time of 86 ns, as derived from the program HYDRONMR.^20^ All NMR data were processed with the nmrpipe/nmrdraw suite of programs.^21^ Figures displaying NMR spectra were prepared using NMRview (onemoonscientific.com), molecular structures were drawn using PyMol (pymol.org).

## Notes

As a service to our authors and readers, this journal provides supporting information supplied by the authors. Such materials are peer reviewed and may be re-organized for online delivery, but are not copy-edited or typeset. Technical support issues arising from supporting information (other than missing files) should be addressed to the authors.
