# Supplementary material for: The Archaeal Exosome: Identification and Quantification of Site-Specific Motions That Correlate with Cap and RNA Binding
Source: Angew Chem Int Ed Engl. 2013 Jun 26;52(32):8312–6. doi: 10.1002/anie.201302811 (PMC3840697; doi:10.1002/anie.201302811)
Supplement: Supplementary file 1 [file anie0052-8312-sd1.pdf]

Angewandte Chemie
Eine Zeitschrift der Gesellschaft Deutscher Chemiker

Supporting Information
© Wiley-VCH 2013
69451 Weinheim, Germany

The Archaeal Exosome: Identification and Quantification of Site-Specific Motions That Correlate with Cap and RNA Binding**

*Maxime J. C. Audin, Georg Dorn, Simon A. Fromm, Kerstin Reiss, Stefan Schütz, Matthias K. Vorländer, and Remco Sprangers**

anie_201302811_sm_miscellaneous_information.pdf

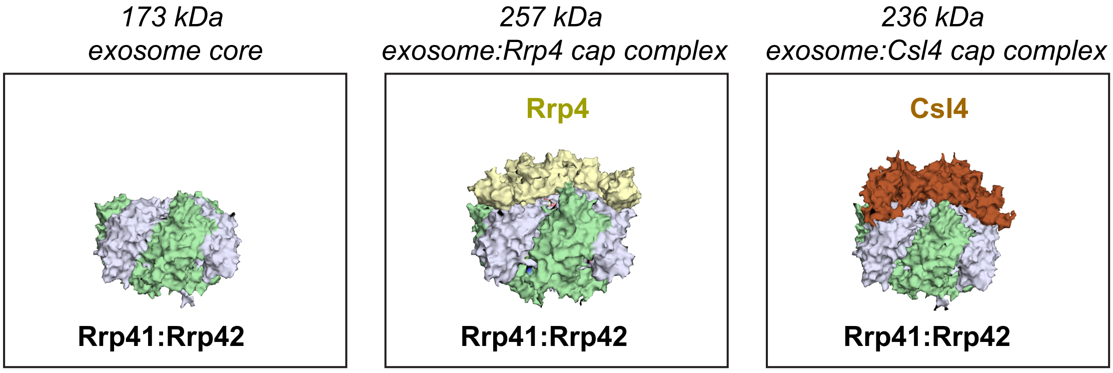

Figure S1. Architecture of the exosome complex. Rrp42 is displayed as a cartoon (green), Rrp41 is shown in gray, Rrp4 in yellow and Csl4 in brown. The displayed structures are based on PDB-entries 2BR2 (exosome core)[1], 2JEA (exosome Rrp4 complex)[2] and 3M7N (exosome Csl4 complex, displayed structure is a homology model based on the *archaeoglobus fulgidus* structure of the complex).[3]

The exosome core (Rrp41:Rrp42) can interact with substrate RNA and degrade this in a processive manner in the 3' to 5' direction. During this process, the exosome does not release the substrate. We thus expect that cap proteins will not be recruited to the processing exosome core: substrate RNA complex. It should be noted that the amounts of cap-free exosome (Rrp41:Rrp42) is expected to be very low in a cellular context as cap proteins, Rrp41 and Rrp42 are present in similar relative amounts.[4]
The interaction between the free exosome and the cap proteins (Csl4 or Rrp4) is very tight. After formation of the exosome:cap complex RNA substrate can be recruited and degraded; the cap proteins will remain bound to the exosome core during this process.

To prepare the samples used in the current study, the *Sulfolobus solfataricus* Rrp41, Rrp42, Rrp4 and Csl4 DNA (a kind gift from E. Conti, MPI Munich) was cloned into pET vectors carrying a TEV cleavable N-terminal His6-tag. Point mutations were introduced using the Quikchange approach (Stratagene). U-[^{2}H, ^{15}N] Ile-δ1, Leu-δ, Val-γ [^{1}H, ^{13}C] labeled Rrp42 proteins were obtained by overexpression of the corresponding gene in BL21(DE3) Codon Plus RIL (Stratagene) cells in 100 % D2O minimal medium, as previously described.[5] Purification of all constructs was achieved by using Ni affinity chromatography followed by cleavage of the histidine tag and size-exclusion chromatography. If required, amide protons of Rrp42 were back-exchanged by refolding the GuHCl denatured protein in H_2O based buffer. Exosome core complexes were reconstituted by combining separately purified components. Exosome-cap complexes were obtained by addition of purified Rrp4 or Csl4 to the exosome core complex. NMR samples contained between 0.05 and 1.5 mM protein (monomer concentration) in 30 mM KPO4 pH 6.8 (or 25 mM Hepes pH 7.5), 150 mM NaCl, 1 mM DTT in 100 % D2O or in 95:5 H2O:D2O. For the exosome RNA complex, excess of RNA was removed by size exclusion chromatography such that one RNA molecule was present per hexameric exosome core complex.

Substrate RNA (20 adenines linked to a hairpin structure) was produced by *in-vitro* transcription using a linearized pSP64 plasmid that contains the substrate RNA followed by a 3' HDV ribozyme that auto-cleaves the RNA cotranscriptionally. The RNA was purified over a Dionex DNAPac PA-100 column at 75 °C using a NaCl gradient in 5 M urea. Substrate RNA was not degraded during NMR experiments due to lack of phosphate in the buffer, the 2',3'-cyclic phosphate at the 3' end of the RNA and the hairpin structure at the 5' end of the RNA.

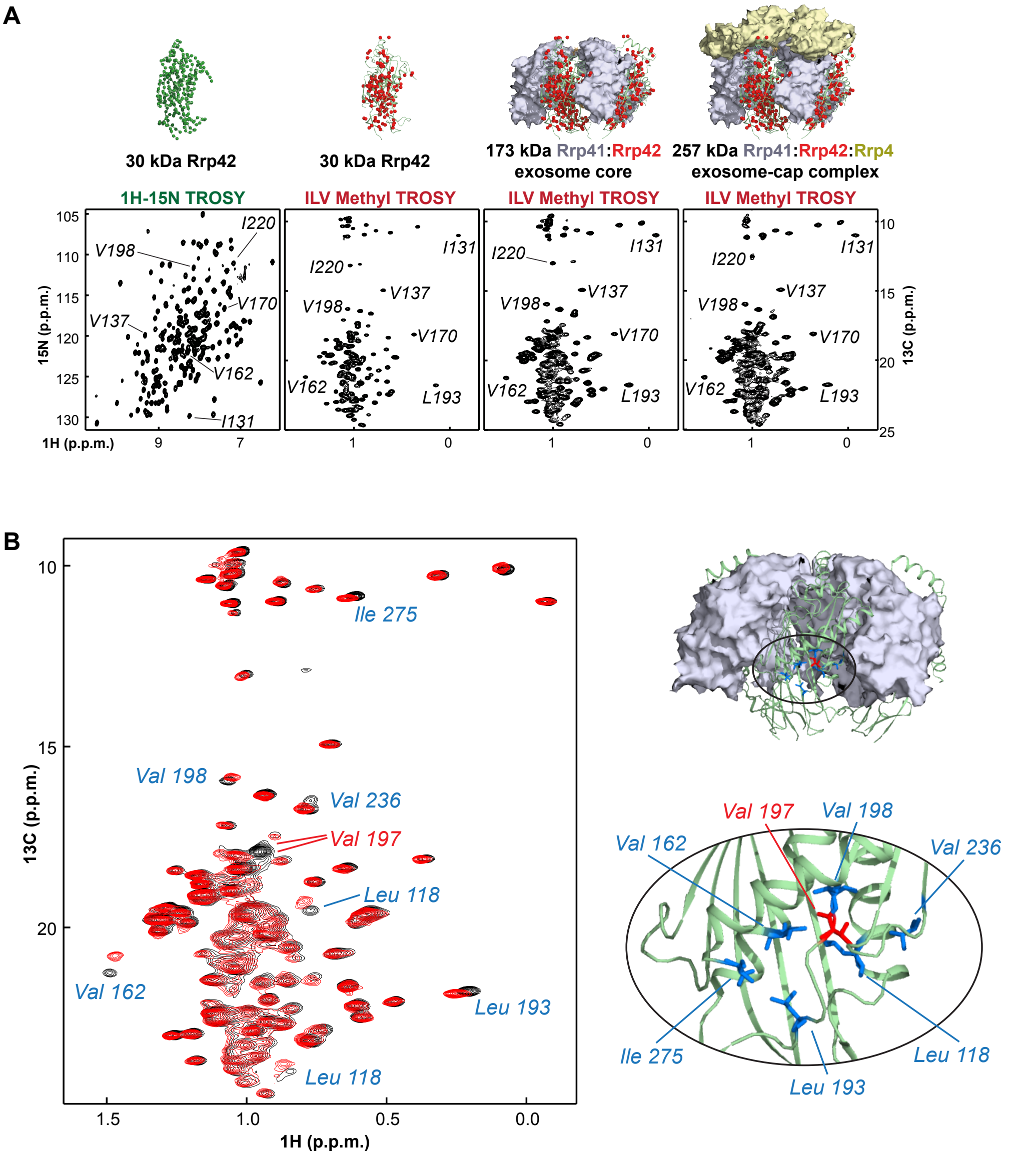

Figure S2. Methyl group assignment strategy. (A) Building blocks used in the divide and conquer approach. First panel: ^{1}H-^{15}N TROSY spectrum of [^{2}H, ^{13}C, ^{15}N] labeled Rrp42 monomer. H-N groups are shown as green spheres. Second panel: ^{1}H-^{13}C methyl TROSY spectrum of U-[^{2}H, ^{15}N] Ile-δ1, Leu-δ, Val-γ [^{1}H, ^{13}C] Rrp42 as monomer. Third panel: Rrp42 within the exosome core complex. Last panel: Rrp42 within the exosome-Rrp4 complex. Labeled methyl groups are shown as red spheres. Exemplary assignments are indicated. Rrp42 backbone sequential assignments were completed using TROSY versions of HNCACB/HNCOCACB experiments. Methyl groups in the Rrp42 monomer were assigned using C(C)(CO)NH TOCSY, H-N-H and H-N-C NOESY spectra. C-C-H HMQC-NOE-HMQC and H-C-H NOE-HMQC spectra were used to assign methyl groups in the exosome-core and exosome-cap complexes. (B) Assignments by point mutations. Left: spectra of WT (black) and V197A (red) exosome complexes. The assignment for V197 is indicated in red. Residues that are in the vicinity of the mutation and that thus experience secondary chemical shift changes are labeled in blue. Right: Location of V197 and the residues that experience secondary chemical shifts on the crystal structure of the exosome core complex.

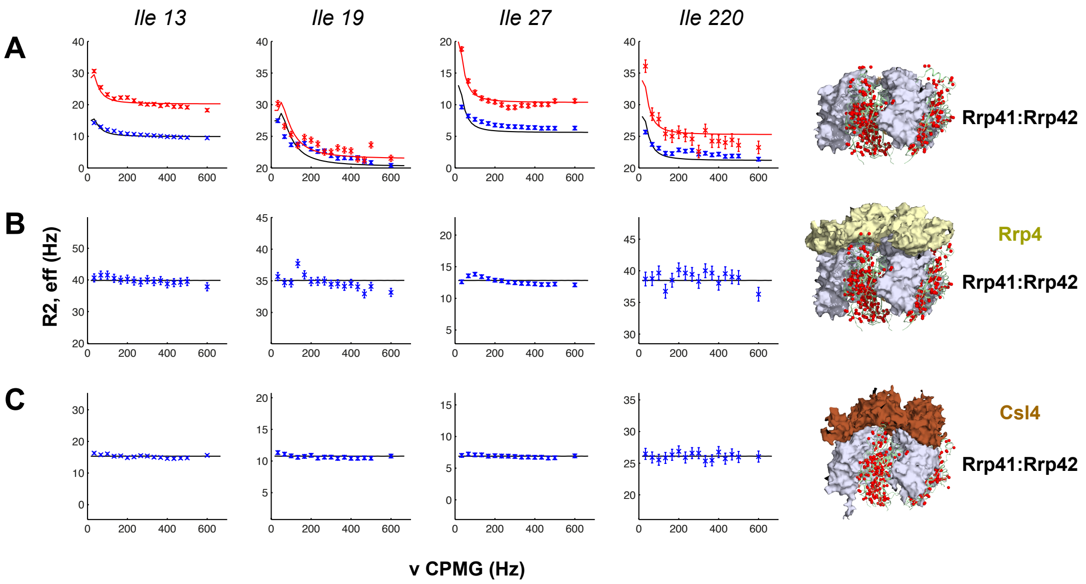

Figure S3. The cap proteins change the dynamics in the exosome core. MQ dispersion profiles observed for Ile 13, 19, 27 and 220. Note that the y-axis has the same range for all graphs for a specific residue to allow for direct comparison of the data. (A) Profiles in the exosome core (identical to Fig 2B in the main text). Blue and red correspond to state A and B respectively. See main text for details. The structure of the exosome core is indicated on the right. (B) Profiles in the exosome-Rrp4 complex. Note that only one state is present in the spectra. The structure of the exosome-Rrp4 complex is shown on the right. (C) Profiles in the exosome-Csl4 complex. Note that only one state is present in the spectra.

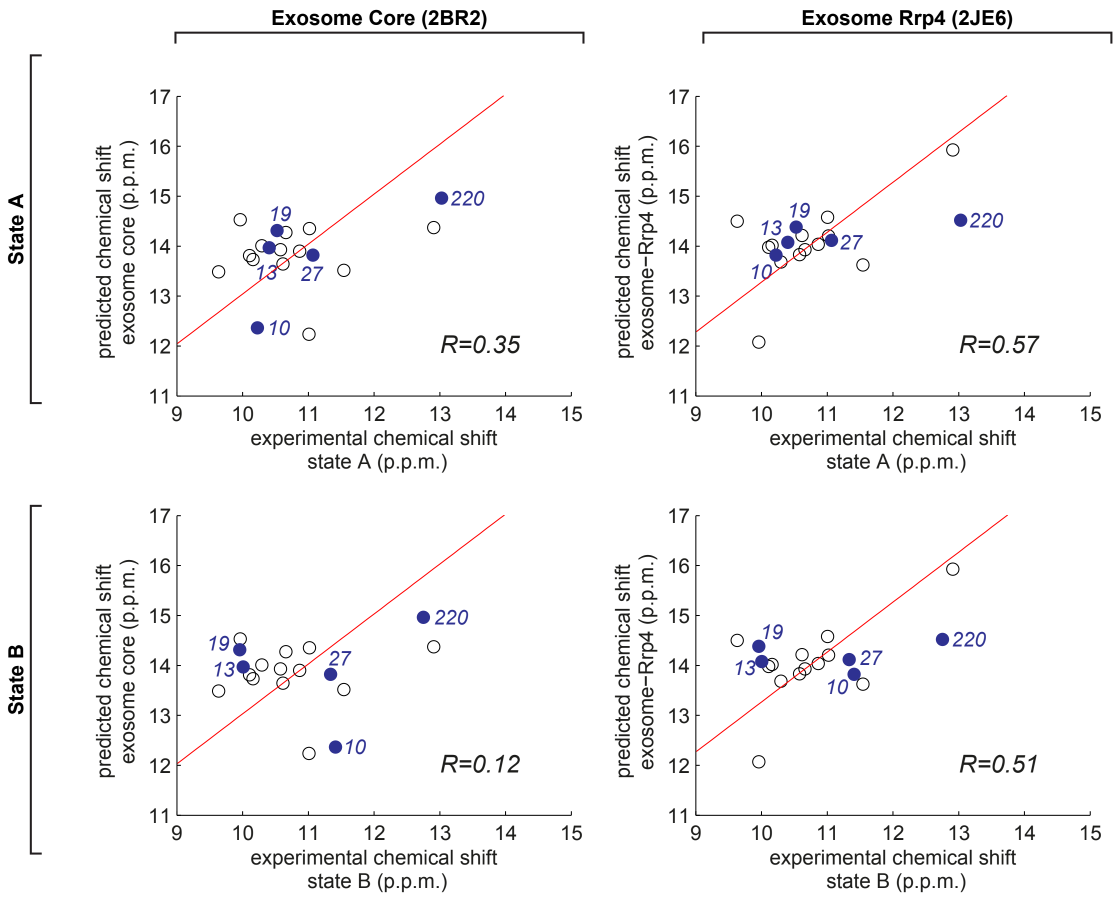

Figure S4. Predicted versus measured chemical shifts for all assigned isoleucine residues. The chemical shifts were predicted using shiftx2[6] using the free exosome complex (2BR2) or the exosome-Rrp4 complex (2JE6) from which Rrp4 was removed as input. The methyl groups that show two conformations have been labeled; the Pearson R correlation coefficient is indicated. A red drawn line indicates the best fit between the predicted and measured shifts (y=x+A), where A corrects for an (potential) offset in chemical shift referencing. Note that none of the correlations is significant, most likely due to the large inaccuracies in the predicted values.

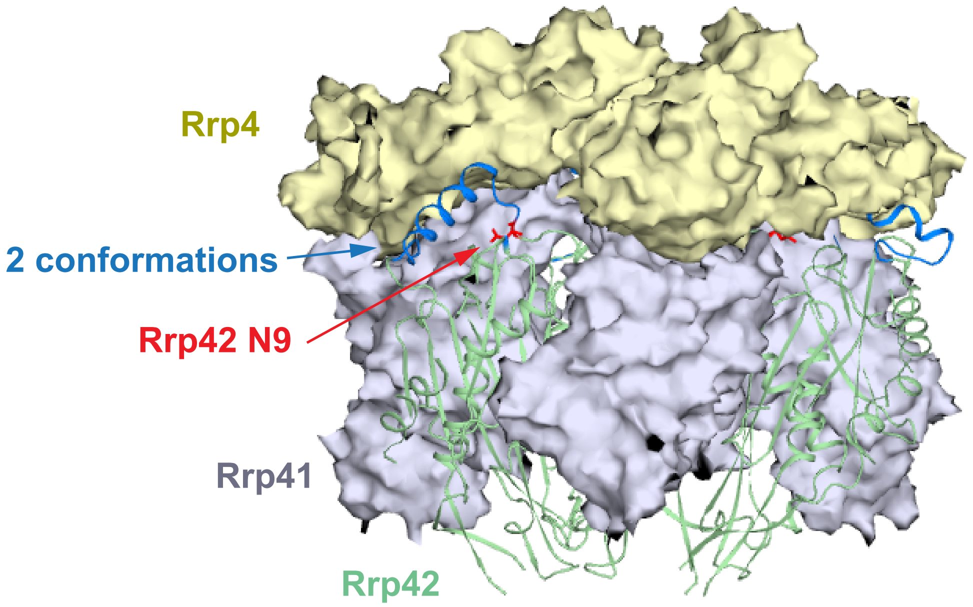

Figure S5. "State A mutant" (N9A) exosome complex. Location of N9 in the exosome complex. N9 is remote from the interaction with the cap structure. Mutations in this residue do thus not change the interaction between the exosome core and the cap directly, but rather indirectly through changes in exosome dynamics.

The identification of the N9A mutant was inspired by the spectra of the assignment mutants (Table S1), where we noticed that the relative intensities of the two sets of peaks varied. This indicated that the equilibrium between the two states could be modified. We then systematically mutated residues that were close, but not directly in the cap-interaction-helix and monitored the state A: state B peak ratio. In this process we identified that the N9A mutation yielded only a single set of resonances.

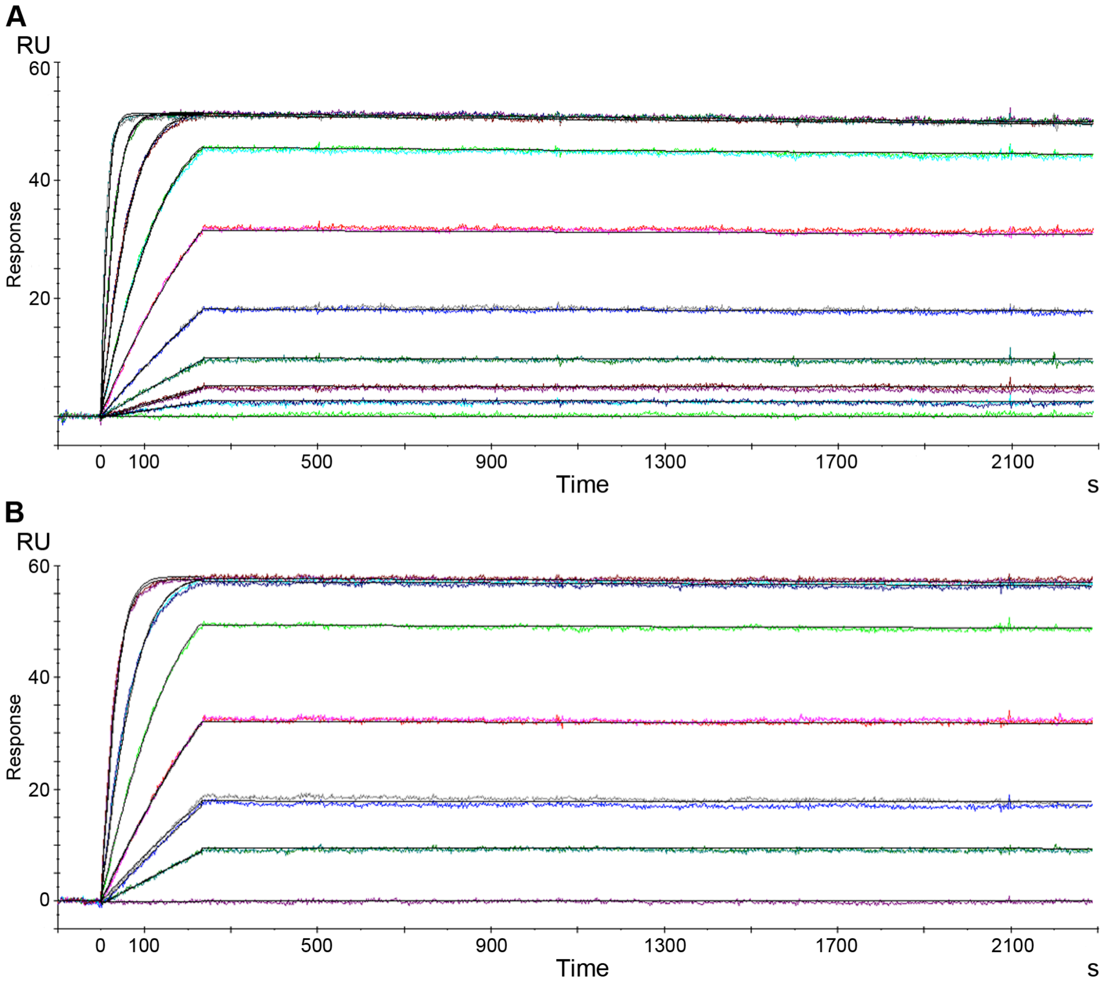

Figure S6 Kinetic SPR analyses of cap protein Rrp4 with His-tagged wildtype (A) or "state A mutant" (B) exosome complex attached to a Ni NTA chip. The double-referenced sensorgrams (indicating that two controls experiment were performed: one without ligand and one without analyte) are overlaid with fits of a "1:1 binding with mass transfer" model.

	wildtype	SE [a]	state A mutant	SE[a]
k_{on} ($M^{-1}s^{-1}$)	$1.7 \cdot 10^6$	$2.1 \cdot 10^3$	$1.7 \cdot 10^6$	$2.8 \cdot 10^3$
k_{off} (s^{-1}) [b]	$< 10 \cdot 10^{-5}$		$< 10 \cdot 10^{-5}$	
**K_D (M)** [c]	**$< 10 \cdot 10^{-11}$**		**$< 10 \cdot 10^{-11}$**	
R_{max} (RU) [d]	51.2	0.01	57.7	0.01
χ^2 (RU^2)	0.17		0.20	

[a] Standard error (obtained from the Biaeval software kit)

[b] The off-rate is at the detection limit of the system

[c] Defined as k_{off} /k_{on}

[d] Theoretical maximum response that is reached when all ligand binding sites are occupied by the analyte; RU refers to response units.

The SPR analyses were performed on a Biacore 2000 system at 15 °C. Two consecutive flow cells (a measurement cell and a reference cell in which no ligand was immobilized) were used. In both the measurement and the reference cell an NTA chip (GE healthcare) was loaded with $NiCl_2$ following the manufacturer's instructions. His-tagged wildtype or "state A mutant" exosome complex was diluted in running buffer (10 mM HEPES, pH 7.4, 150 mM NaCl, 5 % D20,

50 uM EDTA) and 100-110 RU of ligand were non-covalently bound to the experimental flow cell. Untagged cap protein Rrp4 was serially diluted in running buffer to concentrations ranging from 0.23 to 58 nM and injected for 200 s in both the experimental and the reference cell at a flow rate of 50 μl/min. The dissociation phase was followed for 1800 s. Additionally, we recorded a blank curve, where buffer (without analyte) was injected. Both the curve from the reference cell and the blank injection were subtracted from the SPR signal in the measurement cell. To regenerate the surface regeneration buffer (10 mM HEPES, 150 mM NaCl, 0.005% (v/v) P20, 0.35 M EDTA, pH 8.3) was injected for 3 minutes at a flow rate of 20 μl/min in both the reference and the measurement cell.

In addition to the interaction between the exosome complex and the Rrp4 cap we performed experiments to probe for the interaction between the exosome complex and the reduced Rrp4 cap (that lacks one of the domains; See Figure S7). Unfortunately, this protein interacted unspecifically with the sensorchip surface, which resulted in a strong signal from the reference cell. As a consequence, we were not able to extract any reliable interaction data for the exosome: reduced Rrp4 cap complex.

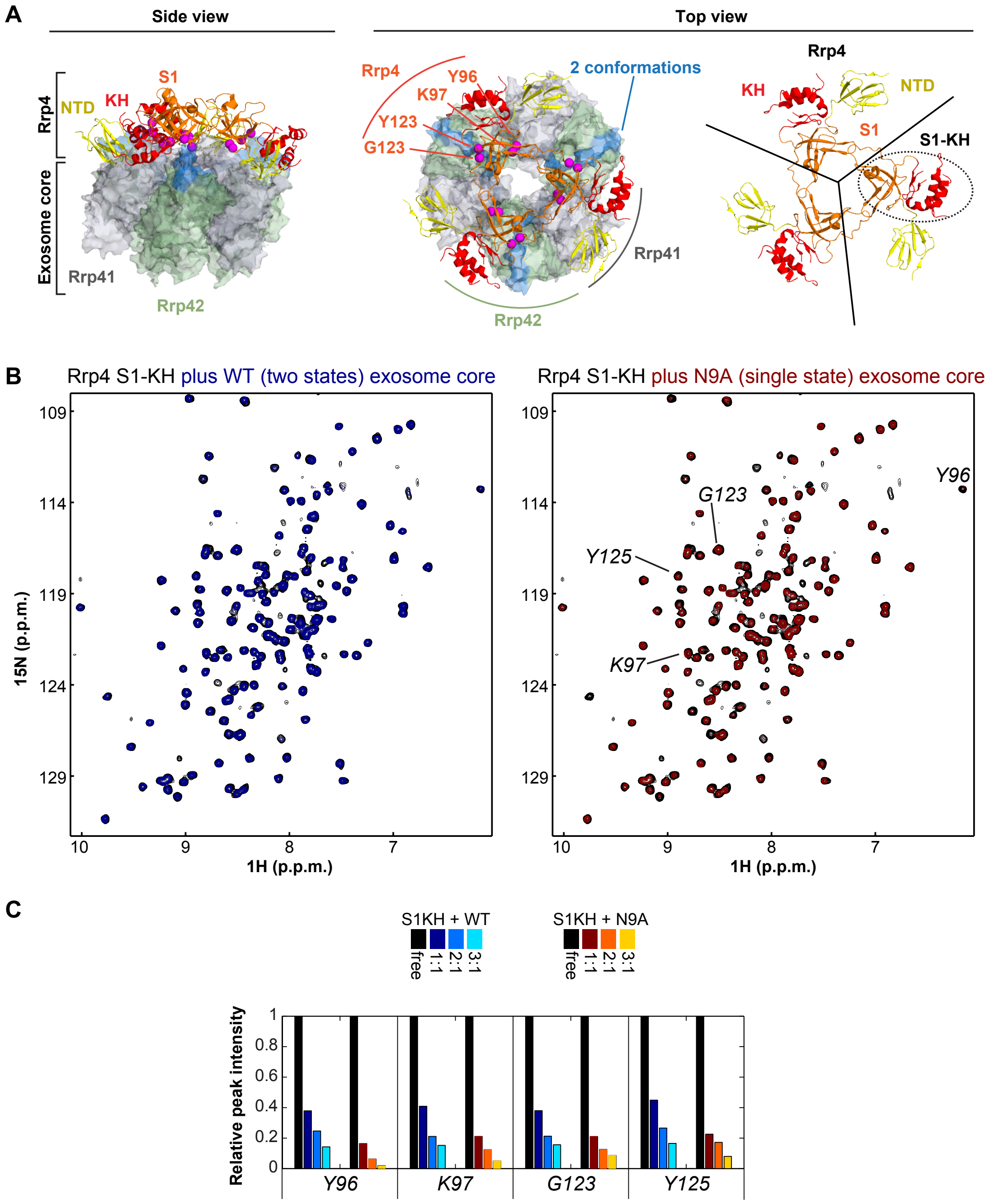

Figure S7 To move the Rrp4 binding affinity into a range where one can discriminate cap binding between WT and "state A mutant" exosome, we deleted one of the three domains from the cap structure (A). This reduced Rrp4 cap contains the domains that interacts with Rrp42 (the S1 and KH domains) but lacks the domain (the NTD; N-terminal domain) that interacts with Rrp41. We then used this reduced Rrp4 cap structure to probe for the interactions with the WT and "state A mutant" exosome. In NMR chemical shift titrations (B), where we added the unlabeled exosome to ^{15}N-labeled reduced cap, we observed a faster decrease in resonance intensity upon addition of the "state A mutant" exosome than upon addition of the WT exosome (C). This implies that the "state A mutant" has a higher affinity for the cap than the WT exosome and establishes that state A plays an important role in the interaction with the Rrp4 cap structure.

(A) Left: Side view of the structure of the exosome-Rrp4 complex. The exosome core is drawn as a surface representation (Rrp41: gray; Rrp42: green), Rrp4 is shown as a ribbon. The three Rrp4 domains are colored separately; NTD (N-terminal domain) in yellow; S1 domain in orange; KH domain in red.

Middle: Top view of the complex, indicating that Rrp42 does not contact the Rrp4 NTD. The region that displays two conformations (blue) contacts both the S1 and KH domains. Selected Rrp4 residues that are in contact with Rrp42 are indicated (see below). Right: Cartoon representation of Rrp4. The S1-KH (reduced Rrp4 protein) region that is used to probe for binding with the exosome is circled.

(B) ^{1}H-^{15}N NMR spectra of the reduced Rrp4 protein in the absence (black) and presence of an equimolar amount of WT exosome (left, blue) or “state A mutant” exosome (red, right). Larger chemical shift perturbations are observed upon addition of the “state A mutant” complex, demonstrating a stronger interaction.

(C) Dependence of reduced Rrp4 peak intensities on the molar excess of exosome (blue-cyan scale) or “state A mutant” exosome (red-yellow scale). Four Rrp4 residues that contact Rrp42 are selected. The signals decrease more rapidly upon addition of the “state A mutant” due to the tighter interaction. Note that the decrease in peak intensity is largely due to fast relaxation in the high molecular weight complex that is formed, preventing accurate extraction of binding constants from the NMR data. After addition of a high excess of (WT or “state A”) exosome the spectra of the reduced cap are no longer visible due to the formation of a large complex.

Residue[1]	Rrp42 monomer		Rrp42 in exosome core		Point mutation[4]
	^{13}C (p.p.m.)	^{1}H (p.p.m.)	^{13}C (p.p.m.)	^{1}H (p.p.m.)	
Ile 10 δ1 (A)[2]	10.349	1.081	10.224	1.050	yes
Ile 10 δ1 (B)	Not applicable[3]		11.412	1.283	yes
Ile 11 δ1	9.836	1.084	9.635	1.026	yes
Ile 13 δ1 (A)	10.394	1.127	10.402	1.153	yes
Ile 13 δ1 (B)	Not applicable		10.004	1.117	yes
Ile 14 δ1	9.972	1.062	10.576	1.079	yes
Ile 19 δ1 (A)	10.265	0.885	10.525	0.879	
Ile 19 δ1 (B)	Not applicable		9.955	0.863	
Val 20 γ1	19.456	1.325	19.86	1.219	
Val 20 γ2	20.208	1.254	19.481	1.296	
Leu 22 δ1	22.933	0.993	22.856	0.98	
Leu 22 δ2	20.245	0.949	20.17	0.942	
Ile 27 δ1 (A)	11.088	1.076	11.066	1.062	yes
Ile 27 δ1 (B)	Not applicable		11.337	1.049	yes
Leu 34 δ1	22.451	1.14	22.385	1.118	
Leu 34 δ2	19.834	0.583	19.668	0.591	
Leu 40 δ1	22.422	1.045	22.356	1.007	
Leu 40 δ2	23.58	1.033	23.504	0.978	
Ile 42 δ1	10.674	0.786	10.659	0.756	
Leu 44 δ1	23.109	1.305	22.995	1.274	
Leu 44 δ2	21.238	1.087	21.066	1.043	
Leu 55 δ1	20.712	0.761	20.731	0.681	
Leu 55 δ2	22.422	0.664	22.084	0.473	
Val 56 γ1	17.994	1.067	17.996	1.042	
Val 56 γ2	19.587	1.04	19.567	1.011	
Leu 58 δ1	---	---	---	---	
Leu 58 δ2	---	---	---	---	
Val 63 δ1	---	---	---	---	yes
Val 63 δ2	---	---	---	---	yes
Leu 64 δ1	21.136	1.165	---	---	
Leu 64 δ2	23.605	1.11	---	---	
Leu 69 δ1	22.232	0.615	22.176	0.603	
Leu 69 δ2	23.035	0.8	23.129	0.772	
Ile 71 δ1	10.394	0.344	10.293	0.317	
Leu 84 δ1	23.372	0.947	---	---	yes
Leu 84 δ2	19.973	0.923	---	---	yes
Ile 85 δ1	10.304	0.23	10.159	0.087	
Val 86 γ1	18.608	1.049	---	---	yes
Val 86 γ2	18.914	1.096	---	---	yes
Val 88 γ1	19.215	1.024	---	---	
Val 88 γ2	18.068	0.996	---	---	
Leu 90 δ1	---	---	---	---	
Leu 90 δ2	---	---	---	---	
Leu 91 δ1	---	---	---	---	

Leu 91 δ2	---	---	---	---	
Leu 93 δ1	---	---	---	---	
Leu 93 δ2	---	---	---	---	
Ile 108 δ1	9.878	1.153	1.123	10.615	yes
Leu 110 δ1	---	---	---	---	yes
Leu 110 δ2	---	---	---	---	yes
Val 113 γ1	19.529	1.046	---	---	yes
Val 113 γ2	20.172	1.215	---	---	yes
Val 114 γ1	19.005	1.036	---	---	yes
Val 114 γ2	19.938	1.023	---	---	yes
Leu 118 δ1	23.912	0.864	24.015	0.856	yes
Leu 118 δ2	19.573	0.81	19.543	0.786	yes
Leu 124 δ1	21.034	0.841	20.822	0.809	
Leu 124 δ2	---	---	---	---	
Leu 126 δ1	23.001	1.205	22.981	1.197	yes
Leu 126 δ2	20.61	0.857	20.554	0.85	yes
Leu 129 δ1	24.014	1.07	23.981	1.045	
Leu 129 δ2	19.804	0.911	19.754	0.908	
Val 130 γ1	19.005	1.061	19.016	1.058	
Val 130 γ2	18.417	1.042	18.379	1.035	
Ile 131 δ1	11.027	-0.082	11.018	-0.067	
Val 137 γ1	19.689	1.018	19.702	1.005	
Val 137 γ2	14.955	0.71	14.945	0.707	
Val 140 γ1	18.184	0.883	18.129	0.871	yes
Val 140 γ2	---	---	---	---	yes
Leu 142 δ1	21.647	0.953	21.474	0.952	
Leu 142 δ2	23.328	0.978	23.246	1.006	
Val 144 γ1	18.637	0.966	---	---	yes
Val 144 γ2	17.527	0.867	---	---	yes
Val 146 γ1	---	---	---	---	yes
Val 146 γ2	---	---	---	---	yes
Leu 147 δ1	23.966	1.049	---	---	yes
Leu 147 δ2	---	---	---	---	yes
Val 153 γ1	---	---	---	---	yes
Val 153 γ2	---	---	---	---	yes
Leu 154 δ1	22.948	1.039	---	---	yes
Leu 154 δ2	---	---	---	---	yes
Leu 159 δ1	23.589	1.042	23.689	1.198	
Leu 159 δ2	21.574	1.133	21.449	1.137	
Val 162 γ1	19.733	1.378	19.821	1.345	
Val 162 γ2	21.222	1.517	21.265	1.502	
Leu 165 δ1	24.657	0.938	24.668	0.937	
Leu 165 δ2	21.267	0.876	21.441	0.86	
Val 170 γ1	18.41	0.657	18.356	0.642	
Val 170 γ2	18.184	0.386	18.138	0.365	
Val 173 γ1	---	---	19.702	1.018	

Val 173 γ2	---	---	19.129	1.152	
Ile 180 δ1	10.997	0.906	11.008	0.899	
Val 182 γ1	19.653	1.274	19.856	1.317	
Val 182 γ2	19.14	1.158	19.106	1.148	
Val 187 γ1	19.683	1.254	19.106	1.158	
Val 187 γ2	---	---	19.585	1.24	
Val 188 γ1	18.785	1.166	18.824	1.162	
Val 188 γ2	16.446	0.945	16.356	0.941	
Leu 191 δ1	21.463	0.773	21.418	0.774	
Leu 191 δ2	24.283	1.01	24.356	1.018	
Leu 193 δ1	21.764	0.16	21.783	0.213	
Leu 193 δ2	21.545	0.673	21.624	0.642	
Val 197 γ1	17.322	1.02	17.904	0.954	yes
Val 197 γ2	18.288	1.055	18.389	1.02	yes
Val 198 γ1	19.719	1.008	19.816	0.97	
Val 198 γ2	16.285	1.082	15.932	1.071	
Ile 200 δ1	9.739	1.061	9.96	1.051	
Val 202 γ1	20.099	1.062	20.237	1.036	
Val 202 γ2	18.552	1.3	18.423	1.253	
Val 205 γ1	17.083	1.125	17.194	1.071	
Val 205 γ2	19.163	1.186	19.147	1.171	
Leu 209 δ1	23.591	1.057	23.697	1.032	
Leu 209 δ2	20.66	0.975	20.254	0.909	
Val 210 γ1	16.637	0.839	16.751	0.8	yes
Val 210 γ2	19.792	0.581	19.637	0.566	yes
Val 211 γ1	---	---	19.504	0.989	yes
Val 211 γ2	---	---	---	---	yes
Leu 215 δ1	---	---	22.561	1.1	yes
Leu 215 δ2	---	---	22.122	1.143	yes
Ile 220 δ1 (A)	13.178	1.06	13.031	1.010	
Ile 220 δ1 (B)	Not applicable		12.753	1.020	
Ile 225 δ1	13.093	0.945	12.911	0.796	
Leu 233 δ1	19.216	0.672	18.774	0.759	
Leu 233 δ2	22.564	0.893	22.668	0.927	
Ile 235 δ1	11.094	1.107	11.543	0.59	yes
Val 236 γ1	17.556	0.975	16.441	0.772	
Val 236 γ2	18.537	1.192	19.566	0.952	
Ile 238 δ1	11.284	1.115	10.106	0.486	yes
Leu 248 δ1	22.292	1.2	---	---	
Leu 248 δ2	20.941	1.14	---	---	
Ile 251 δ1	10.53	1.017	---	---	yes
Val 263 γ1	20.181	1.326	20.106	1.318	
Val 263 γ2	18.537	1.192	18.606	1.184	
Leu 265 δ1	20.508	0.669	19.918	0.625	
Leu 265 δ2	22.422	0.756	22.803	0.733	
Leu 266 δ1	22.82	0.919	22.619	0.881	

Leu 266 δ2	21.659	0.935	22.043	0.93	
Leu 269 δ1	21.151	1.029	20.879	1.029	
Leu 269 δ2	---	---	---	---	
Leu 273 δ1	22.451	0.649	22.502	0.608	
Leu 273 δ2	20.04	0.982	19.957	0.94	
Ile 275 δ1	10.816	0.629	10.867	0.62	

Table S1:
Assigned chemical shifts for Rrp42 as a monomer and in the exosome core.

[1] Leu and Val methyl groups were not stereo-specifically assigned.
[2] A and B refer to the states A and B in the exosome core.
[3] The Rrp42 monomer only displays one state.
[4] Indicated if a point mutation was made to assign (or check the assignment of) the residue.

References

[1] E. Lorentzen, P. Walter, S. Fribourg, E. Evguenieva-Hackenberg, G. Klug, E. Conti, *Nat Struct Mol Biol* **2005**, *12*, 575-581.

[2] E. Lorentzen, A. Dziembowski, D. Lindner, B. Seraphin, E. Conti, *EMBO Rep* **2007**, *8*, 470-476.

[3] S. Hartung, T. Niederberger, M. Hartung, A. Tresch, K. P. Hopfner, *Nucleic Acids Res* **2010**, *38*, 5166-5176.

[4] P. Walter, F. Klein, E. Lorentzen, A. Ilchmann, G. Klug, E. Evguenieva-Hackenberg, *Mol Microbiol* **2006**, *62*, 1076-1089.

[5] V. Tugarinov, V. Kanelis, L. E. Kay, *Nat Protoc* **2006**, *1*, 749-754.

[6] B. Han, Y. Liu, S. W. Ginzinger, D. S. Wishart, *J Biomol NMR* **2011**, *50*, 43-57.
